# Strengthening Antimicrobial Stewardship Programs in Latin America Through Telementoring: Results From the TEACH PROA-ECHO Initiative

**DOI:** 10.1093/ofid/ofag034

**Published:** 2026-01-27

**Authors:** Rodolfo Ernesto Quirós, Javier Santiago Araujo, Alejandra Macchi, Eugenia Di Libero, Miranda Teruel, María Fernanda Maldonado, Ana Carolina Barbosa de Lima, Andrea Zurawski, Jorge Mera

**Affiliations:** PROAnet Organization, Ciudad Autónoma de Buenos Aires, Argentina; Pharmacy Service, Hospital Cuenca Alta Nestor Kirchner, Buenos Aires, Argentina; Infectious Diseases Service, Sanatorio Las Lomas, San Isidro, Buenos Aires, Argentina; Pharmacy Service, Hospital Evita de Lanús, Buenos Aires, Argentina; PROAnet Organization, Ciudad Autónoma de Buenos Aires, Argentina; ECHO Institute-Health Sciences Center at the University of New Mexico, Albuquerque, USA; ECHO Institute-Health Sciences Center at the University of New Mexico, Albuquerque, USA; ECHO Institute-Health Sciences Center at the University of New Mexico, Albuquerque, USA; ECHO Institute-Health Sciences Center at the University of New Mexico, Albuquerque, USA

**Keywords:** antimicrobial stewardship, capacity building, healthcare facility assessment, telementoring, training support

## Abstract

**Background:**

Antimicrobial resistance (AMR) is a major global health threat, with a disproportionate impact on low- and middle-income countries. The TEACH PROA-ECHO (Telementoring, Equity & Advocacy Collaboration for Health through Antimicrobial Stewardship) initiative aimed to strengthen antimicrobial stewardship programs (ASPs) in Latin America using the ECHO telementoring model.

**Methods:**

From March to December 2024, 80 healthcare institutions from 10 Latin American countries participated in biweekly telementoring sessions focused on case-based learning guidance on stewardship interventions and collaborative problem-solving related to antimicrobial prescribing and surveillance. A validated self-assessment tool was used to categorize baseline ASP development and monitor progress over time. Learning activities were evaluated using Moore's framework, and participating teams identified local barriers and enablers for stewardship activities.

**Results:**

Of the 80 institutions, 73 (91%) completed the program. A total of 322 professionals were registered in the project, accounting for 2166 attendances. Moore's indicators showed high satisfaction (Net Promoter Score 79) and significant knowledge improvement after sessions (97.3% vs 81.4%; *P* < .0001). Mean ASP self-assessment scores increased from 53.1 to 64.0 (*P* < .0001). The proportion of institutions with intermediate or advanced ASP development rose from 56.2% to 83.6% (*P* < .001). Higher baseline scores were associated with for-profit institutions, ASP implementation longer than 5 years, and program continuity (lack of ASP interruptions in the last 5 years).

**Conclusions:**

Telementoring is an effective and scalable approach to strengthening antimicrobial stewardship in Latin America. The TEACH PROA-ECHO model represents a valuable strategy to support national AMR action plans in resource-limited settings.

## Teaser

The TEACH PROA-ECHO telementoring project improved antimicrobial stewardship programs in 73 Latin American institutions, significantly raising their development scores, and knowledge of the participants, demonstrating a scalable, effective approach to support national AMR action plans in resource-limited settings.

Antimicrobial resistance (AMR) has emerged as one of the greatest threats to global public health. If effective measures are not implemented, projections indicate that by 2050, infections caused by resistant microorganisms could result in up to 10 million deaths annually, surpassing fatalities from cancer, diabetes, and other chronic diseases [1, 2]. The impact will be particularly significant in low- and middle-income countries (LMICs), with South Asia, Latin America, and the Caribbean considered super-regions with the highest all-age forecasted AMR mortality rate in 2050 [2].

Hospitals in LMICs face context-specific challenges in implementing antimicrobial stewardship programs (ASPs) [3, 4]. In Latin America, the implementation of ASPs remains inconsistent and limited in scope [5]. Key barriers include a lack of training, insufficient administrative support, and limited access to microbiological data [6, 7]. Furthermore, facility ownership status of healthcare facilities has been associated with the degree of development of ASP [6].

To address these gaps, the Telementoring, Equity & Advocacy Collaboration for Health through Antimicrobial Stewardship (TEACH AMS) project was developed. This project is funded by Pfizer and led by the ECHO Institute at the University of New Mexico in partnership with the American Society of Microbiology. The project utilized the ECHO model, a virtual hub-and-spoke approach combining expert led learning with peer-to-peer mentoring [8].

In this article, we describe the design, implementation, and evaluation of the TEACH PROA-ECHO project across ten Latin American countries, with the primary goal of strengthening ASPs in participating healthcare facilities.

## METHODS

### Study Design and Setting

**Figure 1.**
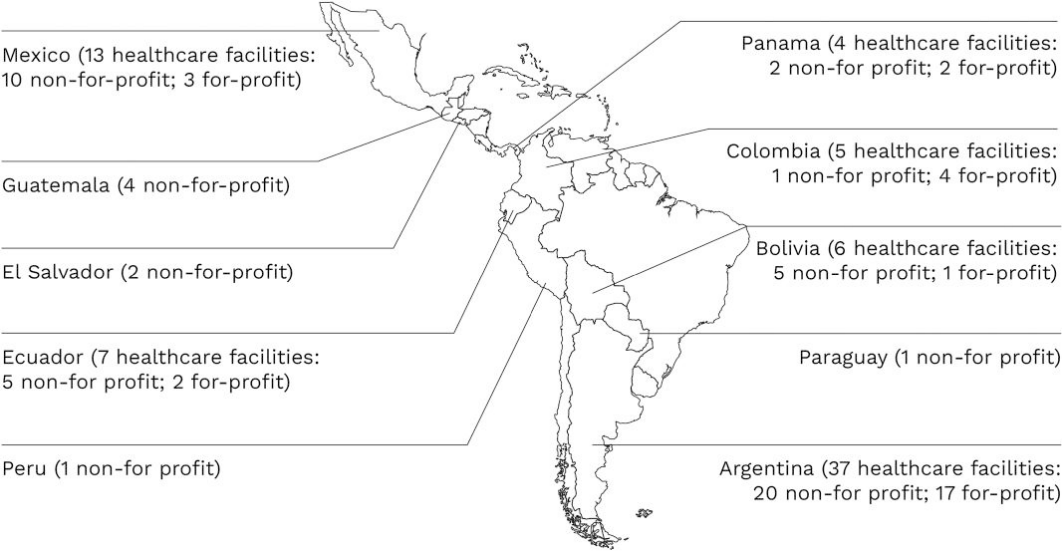
A world map showing the geographic distribution of 80 surveyed healthcare facilities across multiple countries. Facilities are marked and color-coded by ownership status, distinguishing for-profit from not-for-profit hospitals. The map illustrates variation in facility locations and ownership types across regions.

A multicenter, quasi-experimental study was conducted between March and December 2024 in 80 healthcare facilities across Argentina, Mexico, Ecuador, Bolivia, Colombia, Panama, Guatemala, El Salvador, Peru, and Paraguay (Figure 1).

### Participants and Intervention

All 160 hospitals that has used PROAnet (www.proanet.org) were invited to participate. Thos with a multidisciplinary team including infectious diseases physicians, pharmacists and microbiologists were eligible to participate.

The intervention consisted of 18 core sessions and 13 voluntary case-based sessions delivered via the iECHO platform only to the ASP team of each facility, and not to frontline providers. The 18 core sessions incorporated case-based learning activities, guidance on stewardship interventions, and collaborative problem-solving related to antimicrobial prescribing and surveillance (Supplementary Table 1). While the core sessions included a didactic as well as case-based learning, the 13 voluntary sessions were strictly case-based learning.

### Patient Consent Statement

This study does not include variables requiring patient consent. The standard evaluation study, which included web-based program registration and a post-session survey, was reviewed and approved by the Human Research Protection Services at the University of New Mexico under exempt category 2, which pertains to low-risk tests, surveys, interviews, or observations (protocol 20-469). Ethical approvals for the entire study, including standard evaluation instruments, facility self-assessments, and follow-up surveys, varied by country and were obtained by participating healthcare institutions on an individual basis.

### Evaluation Tools

Baseline level and progress of ASP development was evaluated using a self-assessment instrument adapted from different tools and previously validated in a study conducted across 20 healthcare facilities in 5 Latin American countries [6, 9–19]. Using this instrument, a previous study showed that a higher level of ASPs development was significantly associated with better indicators of antimicrobial use and consumption and a lower incidence density of infections by multidrug-resistant microorganisms [20]. The instrument consists of 94 indicators, grouped into 53 blocks, 14 components, and 5 domains (administrative support, clinical guidelines, prescribing optimization, monitoring, and education). Each indicator is rated based on total, partial, or noncompliance with each activity listed. Verifier items were included for each indicator to facilitate the rating and reduce the subjectivity of the ASP team members. Data were collected at baseline, prior to the intervention, and post-intervention using a free web platform (www.proanet.org). While the score of each component can range from 0 to 100, the score of each domain is the average of the components that comprise it. The overall score is the average of the scores of all domains. Considering the overall score, ASPs were classified into four levels of development: *inadequate* (0–25 points), *basic* (26–50 points), *intermediate* (51–75 points), and *advanced* (76–100 points). Institutions were stratified into 2 groups based on their baseline self-assessed ASP development score: inadequate or basic (≤51) and intermediate or advanced (>51).

Progress across levels of learning was evaluated using Moore's level outcome framework, which evaluates progress from participation and knowledge gains to changes in clinical performance and system level impact [21]. After each session, an anonymous survey, hosted on the iECHO web platform, was shared with participants. The post-session survey includes self-assessment questions on participation, session satisfaction, learning, knowledge, competence, and change in practice. Satisfaction was evaluated as how likely the participant recommends this session to a colleague, using the Net Promoter Score as the difference between the percentage of promotors and detractors [22].

In addition, 2 follow-up surveys were conducted during the project (June and November 2024) to evaluate the impact of the project on different aspects of the development, implementation, and monitoring of the ASPs in the participating healthcare facilities. These surveys were completed jointly by the ASP teams, with only one survey recorded per facility each month. Answers reflected the ASP Team's perception, not that of the frontline providers.

In addition, as part of the case-based learning process, participating ASP teams were also asked to identify factors that facilitated or hindered the implementation of specific stewardship interventions at their institutions as part of the telementoring program. Participants verbalized their comments, and the answers were classified into themes by the authors. These qualitative inputs were categorized as potential enablers or barriers to ASP implementation, and all reported factors were systematically documented for analysis.

### Statistical Analysis

Continuous variables were reported as mean ± standard deviation (SD) or median and interquartile ranges, as appropriate, according to the distribution of variables. We used the Student’s *t*-test for continuous variables and the χ^2^ statistic for qualitative variables. Results were expressed as differences in means or percentages, along with their respective 95% confidence intervals (CIs). The results of the self-assessment are shown as mean ± SD and as percentiles to facilitate comparisons between centers.

A paired Student’s *t*-test was used to compare baseline and final scores. To identify institutional characteristics (eg, for-profit and not-for-profit institutions, number of beds, academic or nonacademic, full-time infectious disease specialist, full-time clinical pharmacist, full-time infection control physician, full-time microbiologist, Infection Control Committee, Pharmacy Committee, time of ASPs implementation prior to project initiation, continuity of the ASP since its implementation) associated with the level of the ASP, a univariate analysis was conducted using the baseline self-assessment score as the outcome. Statistically associated variables (*P* < .10) were introduced into a stepwise multiple linear regression model, and only those that were significantly associated (*P* < .05) remained in the model.

A *P* < .05 (2-tailed) was considered statistically significant. For statistical analyses, we used SPSS version 22 software (IBM, Chicago, IL).

## RESULTS

### Participation and Engagement

A total of 322 professionals registered in the project contributing 2166 attendances. Physicians represented the largest group (34.4%), followed by pharmacists (23.5%), microbiologists (18.5%), and other healthcare workers (23.6%). The median participation per health facility was 3 (percentiles 25: 3; percentiles 75: 4). A total of 1448 (66.8%) attendances were recorded in the core sessions and 718 (33.2%) in the case presentation sessions. Post-session survey response rates were 60.4% and 58.3%, respectively.

### Impact on ASP Development Measured by Self-assessment

Of the 80 enrolled institutions, 73 (91.3%) completed both baseline and final self-assessment scores. The average global self-assessment score increased significantly from 53.1 to 64.0 (mean diff. 10.9; 95% CI 8.0–13.7; *P* < .0001) (Table 1). Improvements were observed across all domains, with the largest increase in education, training, and safety climate (54.5 vs 39.0; diff. 15.5; 95% CI 11.3–19.5; *P* < .0001) (Table 1). The percentage of institutions classified as having intermediate or advanced ASP development increased from 56.2% at the beginning of the project to 83.6% at the end (diff. 27.4%; 95% CI 13.2%–41.6%; *P* = .0006).

**Table 1.** Baseline and Final Self-Assessment by Domains and Overall Score of the 73 Healthcare Facilities

Domains	Baseline Self-Assessment	Final Self-Assessment	Difference	95% CI	*P* ^a^	% Variation^b^
Administrative support, ASP team, and infrastructure	55.8 ± 8.6	65.2 ± 17.7	9.4	5.8–12.9	<.0001	17%
Clinical guidelines	57.6 ± 27.9	67.7 ± 24.5	10.1	5.5–14.6	<.0001	18%
Strategies to optimize antimicrobial prescribing	61.9 ± 20.0	70.9 ± 19.2	9.0	5.5–12.5	<.0001	15%
Monitoring and reporting	51.1 ± 20.6	61.7 ± 19.1	10.6	6.4–14.6	<.0001	21%
Education, training, and safety climate	39.0 ± 21.7	54.5 ± 22.1	15.5	11.3–19.5	<.0001	40%
Global score	53.1 ± 16.6	64.0 ± 16.4	10.9	8.0–13.7	<.0001	21%

^a^Paired Student’s *t*-test.

^b^Percentage variation final versus baseline score.

At baseline, for-profit healthcare facilities had a higher percentage of advanced ASPs compared to not-for-profit institutions (20.7% vs 5.9%; diff. 14.8%; 95% CI 0.4%–29.2%; *P* = .0653). They also scored higher across most domains, with significant differences in clinical guidelines, and strategies to optimize antimicrobial prescribing (Supplementary Table 2).

Both facility types improved similarly (10.2 and 11.2 mean differences, respectively), while facilities with inadequate or basic ASPs at baseline demonstrated greater gains than those with intermediate or advanced ASPs (14.2 and 7.8 mean differences, respectively).

In the univariate analysis, variables associated with higher ASP development included for-profit status (57.7 vs 49.5; mean difference 8.2; 95% CI 0.6–15.8; *P* = .0345), longer ASP implementation duration (not implemented 38.7; ≤ 5 years 52.0; >5 years 63.6; *P* < .0000), and greater continuity of ASP activities (prolonged interruptions 44.2; short interruptions 54.5; no interruptions 64.7; *P* = .0003) (Table 2). These variables remained independently associated with baseline ASP development in the multivariate analysis (Table 3).

**Table 2.** Univariate Analysis. Association of ASP Global Score With Institutional Characteristics

Healthcare Facility Characteristics	Overall Mean ± DS	Difference	95% CI	*P*
Hospital ownership status^a^				
For-profit (n = 29)	57.7 ± 16.3	8.2	0.6–15.8	.0345
Not-for-profit (n = 51)	49.5 ± 16.6			
Institution affiliation^a^				
Academic (n = 64)	52.6 ± 17.3	0.6	−8.9–10.0	.9027
Nonacademic (n = 16)	52.0 ± 15.6			
Number of beds^a^				
≤110 beds (n = 25)	55.5 ± 17.0	4.4	−3.7–12.5	.2828
>110 beds (n = 55)	51.1 ± 16.8			
Residences^a^				
No (n = 14)	54.1 ± 13.8	1.9	−8.0–11.8	.7036
Yes (n = 66)	52.2 ± 17.5			
Infectious disease residency^a^				
If (=21)	53.9 ± 19.3	2.5	−6.8–11.8	.5899
No (n = 45)	51.4 ± 16.8			
Pharmacy residence^a^				
If (=20)	53.2 ± 20.2	1.5	−7.9–11.0	.7493
No (n = 46)	51.7 ± 16.4			
Pharmacy committee^a^				
If (=53)	52.8 ± 18.0	0.9	−7.0–8.9	.8163
No (n = 27)	51.9 ± 14.6			
Frequency of meetings of the pharmacy committee^a^				
≥6 meetings/year (n = 33)	52.5 ± 20.2	−0.8	−11.1–9.6	.8864
<6meetings/year (n = 20)	53.3 ± 14.2			
Infection control committee^a^				
If (=75)	53.1 ± 16.3	10.1	−5.3–25.6	.1954
No (n = 5)	43.0 ± 24.0			
Frequency of meetings of the infection control committee^a^				
≥6 meetings/year (n = 60)	53.0 ± 16.0	−0.7	−10.4–8.8	.8842
<6 meetings/year (n = 15)	53.7 ± 18.0			
Length of ASP implementation prior to project initiation^b^				
Not implemented (n = 17)	38.7 ± 16.7			.0000
≤5 years (n = 40)	52.0 ± 12.5			
>5 years (n = 23)	63.6 ± 16.3			
Continuity of the ASP since its implementation^b^				
Prolonged interruptions (>1 year) (n = 11)	44.2 ± 13.3			.0003
Short interruptions (≤1 year) (n = 30)	54.5 ± 11.8			
No interruptions (n = 22)	64.7 ± 15.1			

^a^Student's *t*-test for independent samples.

^b^ANOVA test.

**Table 3.** Multiple Linear Regression Analysis

Variable	Coefficient	Standard Error	T	*P*
For-profit healthcare facility	6.84	3.30	2.08	.0424
ASP implemented for >5 years^a^	9.21	3.25	2.84	.0063
Short interruptions of the ASP (≤1 per year)^b^	11.47	4.27	2.69	.0094
ASP without interruptions^c^	18.96	4.47	4.24	.0001

^a^Variable not retained in the model: implementation time ≤5 years.

^b^Reference variable for implementation time: not having ASP implemented.

^c^Reference variable for ASP continuity: prolonged interruptions of ASP > 1 per year.

### Impact on Levels of Learning Measured Through Moore's Outcomes Framework

Post-session evaluation surveys demonstrated high levels of satisfaction (NPS 79) and increasing knowledge after each session (97.3% vs 81.4%; diff.15.9%; 95% CI 13.6%–18.2%; *P* < .0000). Across sessions, more than 85% of respondents reported the content as highly relevant and reported plans to apply new practices (Table 4).

**Table 4.** Cumulative Moore's Levels From Both Type of Sessions

Moore's Levels	Core Sessions (n = 875)	Case Presentation Sessions (n = 419)	Global (n = 1294)
How relevant was the session to your current work? (as very or extremely relevant)	87.3%	85.2%	86.6%
Knowledge level before the session (as moderately to extremely knowledgeable)	79.4%	85.4%	81.4%
Knowledge level after the session (as moderately to extremely knowledgeable)	97.6%	96.9%	97.3%
You will use what you learned in the sessions for your work? (probably or definitively yes)	93.2%	92.6%	93.0%
Satisfaction level as % of promotors	80.3%	83.3%	81.3%
Satisfaction level as Net Promoter Score	78	82	79

Comparing follow-up surveys between November and June 2024, a significant increase in implementation or modification of ASP strategies was observed (86.3% vs 95.9%; difference 9.6%; 95% CI 0.5%–18.8%; *P* = .0389) (Supplementary Table 3).

Significant improvements were reported in surgical prophylaxis, guidelines for the management of common and/or multidrug-resistant microorganisms, restrictive and/or persuasive control strategies, de-escalation practices, education and training, and measurement of the use and consumption of antimicrobial use (Supplementary Table 4).

While changes in daily clinical practices were modest, significant improvements were observed only in the appropriate use of antimicrobials when indicated and in the use of narrower spectrum antibiotics when appropriate (Supplementary Table 5).

### Perceptions of Enablers and Barriers of ASP Interventions

Across 73 facilities, participants identified 22 enablers and 21 barriers to ASP implementation. The most frequently reported enablers included teamwork (16%), institutional support (12%), implementation of an improvement process (9%), and effective communication (7%). Common reported barriers included limited human resources (14%), resistance to change (12%), lack of training (11%), and restricted access to information or technology (9%). Additional details appear in Supplementary Tables 6 and 7.

## DISCUSSION

The multi-institutional TEACH PROA-ECHO project demonstrated measurable improvements in ASP development and implementation across a diverse network of healthcare facilities. The high level of engagement manifested by 91.3% of institutions completing both baseline and final self-assessment demonstrates that telementoring is a feasible, scalable, and impactful strategy for strengthening ASPs in Latin America.

As the program focused on providing practical tools that ASP teams could implement in their healthcare facilities, participating institutions demonstrated significant improvements in ASP development across all core domains. Although the magnitude of improvement was modest overall (15%–21%), the domain of Education, Training, and Safety Climate showed a markedly greater increase (40%), as expected for a training-centered initiative. This finding underscores the central role of knowledge dissemination and team engagement as key drivers of ASP advancement. Notably, these domains had been consistently identified as deficient in previous regional assessments [6, 7, 23].

The significant improvement in ASP scores among institutions with initially lower baseline development suggests that telementoring may be especially valuable for facilities with limited resources or minimal prior experience in ASP. This model's effectiveness lies in its ability to adapt to various healthcare settings and its capacity to promote equitable knowledge sharing. By fostering interdisciplinary collaboration and sustained engagement, telementoring can help bridge gaps in expertise and capacity, an assertion supported by high Net Promoter Scores and consistent learning gains reported across participant groups.

Our findings mirror prior literature showing persistent barriers to ASP implementation in LMICs, including limited trained personnel, weak institutional support, inadequate information technology infrastructure, and restricted access to microbiological and epidemiological data [6, 7, 24]. This highlights the need for scalable, low-cost strategies such as telementoring.

The association between ASP development and institutional characteristics, particularly for-profit status, longer program duration and continuity, points to operational enablers previously linked to stewardship success, such as sustained leadership engagement and long-term institutional commitment [6, 24]. The comparatively stronger performance of for-profit facilities may reflect more structured professional education and training [25], whereas not-for-profit facilities more frequently face resource constraints, staff resistance and limited information systems [6, 7, 24].

Similar barriers and enablers have been described across LMICs [4], including shortages in human and laboratory resources, limited leadership involvement, and lack of guidelines, while successful programs benefit from standardized protocols, multidisciplinary ASP teams, and timely microbiology data. Despite limited awareness, attitudes toward stewardship are generally positive, suggesting opportunities for capacity building.

A recent survey of healthcare workers in Latin America showed low familiarity with stewardship concepts and limited access to guidelines and antibiograms but willingness to engage in education and feedback, underscoring the potential to strengthen stewardship through improved training, resources, and collaboration [3].

Notably, the TEACH PROA-ECHO model was well received by healthcare workers, as evidenced by high satisfaction rates, and effectively addressed several key ASP implementation gaps. Through interactive case-based learning, peer support, and remote expert mentorship, the model facilitated both knowledge transfer and practical application. This approach led not only to improved ASP development scores but also to self-reported behavioral changes among participants, including increased adoption of best practices, such as appropriate surgical prophylaxis, guideline-based management of common infections, and implementation of prospective audit with feedback and de-escalation strategies.

Nonetheless, several limitations should be acknowledged. As a quasi-experimental design without a control group, the observed improvements cannot be attributed exclusively to the intervention. Participation bias is also a consideration as facilities that were more motivated or better resourced may have been more likely to engage more actively. Additionally, although self-assessments tools provide valuable insights into program development, they are inherently subject to perception bias even when supplemented by objective items as verifiers. Finally, the impact of the program on objective clinical outcomes was not assessed, and some findings, such as participants' perceived changes in antibiotic use, were not independently validated, underscoring the need for further clinical outcomes evaluation.

Despite these limitations, these findings are encouraging and support the value of integrating telementoring as a core component of national action plans on AMR, particularly in LMICs. Building on the TEACH-PROA results, our team is developing a second-generation telementoring initiative, focused on tailored capacity building at the hospital level across Latin America through the implementation of specific quality improvement projects related to antimicrobial stewardship initiatives. Future efforts should prioritize the sustainability of such models, integration with electronic health record systems, and expansion into other key domains of AMR, including infection prevention and control, and diagnostic stewardship. These directions will be essential for achieving long-term system-wide impact.

In summary, the TEACH PROA-ECHO project effectively enhanced ASP development across healthcare facilities in Latin American. By leveraging the ECHO telementoring model, the initiative enabled scalable capacity building, facilitated knowledge transfer, and promoted meaningful behavior change among healthcare workers. This approach represents a practical and adaptable strategy that should be considered for integration into national and regional action plans to strengthen ASPs and combat AMR.

## Supplementary Material

ofag034_Supplementary_Data

## Supplementary Data

Supplementary materials are available at *Open Forum Infectious Diseases* online. Consisting of data provided by the authors to benefit the reader, the posted materials are not copyedited and are the sole responsibility of the authors, so questions or comments should be addressed to the corresponding authors.

## References

[1] O’Neill J . Tackling drug-resistant infections globally: final report and recommendations. The Review on Antimicrobial Resistance **2016**.

[2] Naghavi M, Murray CJ, Kyu HH, et al Global burden of bacterial antimicrobial resistance 1990–2021: a systematic analysis with forecasts to 2050. Lancet 2024; 404:1199–226. 39299261 10.1016/S0140-6736(24)01867-1 PMC11718157

[3] Pauwels I, Versporten A, Ashiru-Oredope D, et al Implementation of hospital antimicrobial stewardship programmes in low- and middle-income countries: a qualitative study from a multi-professional perspective in the Global-PPS network. Antimicrob Resist Infect Control 2025; 14:26. 40188146 10.1186/s13756-025-01541-6 PMC11972458

[4] Harun MGD, Sumon SA, Hasan I, Akther FM, Islam MS, Anwar MMU. Barriers, facilitators, perceptions and impact of interventions in implementing antimicrobial stewardship programs in hospitals of low-middle and middle countries: a scoping review. Antimicrob Resist Infect Control 2024; 13:8. 38263235 10.1186/s13756-024-01369-6 PMC10804809

[5] Hegewisch-Taylor J, Dreser-Mansilla A, Romero-Mónico J, Levy-Hara G. Antimicrobial stewardship in hospitals in Latin America and the Caribbean: a scoping review. Rev Panam Salud Publica 2020; 44:e68. 32973908 10.26633/RPSP.2020.68 PMC7498295

[6] Fabre V, Secaira C, Cosgrove SE, et al Deep dive into gaps and barriers to implementation of antimicrobial stewardship programs in hospitals in Latin America. Clin Infect Dis 2023; 77(S1):S53–61. 37406044 10.1093/cid/ciad184 PMC10321692

[7] Howard P, Pulcini C, Levy Hara G, et al An international cross-sectional survey of antimicrobial stewardship programmes in hospitals. J Antimicrob Chemother 2015; 70:1245–55. 25527272 10.1093/jac/dku497

[8] Arora S, Thornton K, Komaromy M, Kalishman S, Katzman J, Duhigg D. Demonopolizing medical knowledge. Acad Med 2014; 89:30–2. 24280860 10.1097/ACM.0000000000000051

[9] Centers for Disease Control and Prevention (CDC) . The core elements of human antibiotic stewardship programs in resource-limited settings: national and hospital levels. Atlanta: US Department of Health and Human Services; **2018**. Available at: https://www.cdc.gov/antibiotic-use/healthcare/pdfs/stewardship-resource-limited-508.pdf

[10] World Health Organization . Antimicrobial stewardship programmes in health-care facilities in low- and middle-income countries: a WHO practical toolkit. **2020**. Available at: https://apps.who.int/iris/handle/10665/335947 10.1093/jacamr/dlz072 PMC8210188 34222945

[11] Pan American Health Organization . Recommendations for impementing antimicrobial stewardship programs in Latin America and the Caribbean: manual for public health decision-makers. Washington, D.C.; Florida International University; **2018**. Available at: https://iris.paho.org/handle/10665.2/49645

[12] Centers for Disease Control and Prevention (CDC) . Core elements of hospital antibiotic stewardship programs. Atlanta: US Department of Health and Human Services; **2014**. Available at: http://www.cdc.gov/getsmart/healthcare/implementation/core-elements.html

[13] Hénard S, Rahib D, Léon L, et al Antimicrobial consumption reported through standardized reports on infection control activities, relationship with the ICATB public reporting indicator. Med Mal Infect 2011; 41:197–205. 21195568 10.1016/j.medmal.2010.11.013

[14] Direction générale de l’offre de soins . Fiche descriptive de l’indicateur composite de bon usage des antibiotiques (ICATB.2). Paris: DGOS; **2014**.

[15] Centers for Disease Control and Prevention (CDC) . Core elements of hospital antibiotic stewardship programs. Atlanta: US Department of Health and Human Services; **2019**. Available at: https://www.cdc.gov/antibiotic-use/healthcare/pdfs/hospital-core-elements-h.pdf

[16] Pollack LA, Plachouras D, Sinkowitz-Cochran R, et al A concise set of structure and process indicators to assess and compare antimicrobial stewardship programs among EU and US hospitals: results from a multinational expert panel. Infect Control Hosp Epidemiol 2016; 37:1201–11. 27418168 10.1017/ice.2016.115 PMC6533629

[17] World Health Organization Regional Office for Europe . Antimicrobial stewardship interventions: a practical guide. Copenhagen; **2021**.

[18] Centers for Disease Control and Prevention (CDC) . Global Antibiotic Stewardship Evaluation Tool (G-ASET) for inpatient healthcare facilities. Atlanta: CDC; **2024** [cited 2025 Jul 2]. Available at: https://www.cdc.gov/international-infection-control/media/pdfs/Global-Stewardship-Tool-508.pdf

[19] Fabre V, Cosgrove SE, Lessa FC, et al Antibiotic use in medical-surgical intensive care units and general wards in Latin American hospitals. Open Forum Infect Dis 2024; 11:ofae620. 39494448 10.1093/ofid/ofae620 PMC11530953

[20] Quirós RE, Bardossy AC, Angeleri P, et al Antimicrobial stewardship programs in adult intensive care units in Latin America: implementation, assessments and impact on outcomes. Infect Control Hosp Epidemiol 2022; 43:181–90. 33829982 10.1017/ice.2021.80

[21] Moore DE Jr, Green JS, Gallis HA. Achieving desired results and improved outcomes: integrating planning and assessment throughout learning activities. J Contin Educ Health Prof 2009; 29:1–15. 19288562 10.1002/chp.20001

[22] Reichheld FF . The one number you need to grow. Harv Bus Rev 2003; 81:46–54. 14712543

[23] Fabre V, Cosgrove SE, Lessa FC, et al Knowledge, attitudes and perceptions of Latin American healthcare workers relating to antibiotic stewardship and antibiotic use: a cross-sectional multi-country study. Antimicrob Resist Infect Control 2024; 13:47. 38664757 10.1186/s13756-024-01400-w PMC11045452

[24] Nassar H, Abu-Farha R, Barakat M, Alefishat E. Antimicrobial stewardship from health professionals’ perspective: awareness, barriers, and level of implementation of the program. Antibiotics 2022; 11:99. 35052979 10.3390/antibiotics11010099 PMC8773352

[25] Mlambo M, Silén C, McGrath C. Lifelong learning and nurses’ continuing professional development, a metasynthesis of the literature. BMC Nurs 2021; 20:62. 33853599 10.1186/s12912-021-00579-2 PMC8045269

