# Supplementary material for: Strengthening Antimicrobial Stewardship Programs in Latin America Through Telementoring: Results From the TEACH PROA-ECHO Initiative
Source: Open Forum Infect Dis. 2026 Jan 27;13(2):ofag034. doi: 10.1093/ofid/ofag034 (PMC12911034; doi:10.1093/ofid/ofag034)
Supplement: ofag034_Supplementary_Data [file ofag034_supplementary_data.docx]

**Supplementary materials**

**Supplementary - Table 1.** Issues and content of core sessions

| **Issues** | **Contents** |  |  |
| --- | --- | --- | --- |
| - *Antimicrobial resistance: "One Health" concept.* | - Epidemiology and evolution of antimicrobial resistance Relationship to antimicrobial consumption - Public health impact of antimicrobial resistance |  |  |
| - *Principles and generalities of an Antimicrobial Stewardship Program (ASP)* | - Steps to implement an ASP - ASP Self-assessment - Role of the ASP Team Members - Identifying improvement opportunities through a priority matrix |  |  |
| - *Integration of an ASP and an Infection Control and Prevention Program* | - Components of an Infection Control and Prevention program - Interaction with an ASP |  |  |
| - *Mechanisms of action and resistance to antimicrobial s* | - Mechanisms of action of antimicrobial agents Mechanisms of resistance |  |  |
| - *Diagnostic optimization* | - Pre-analytical, analytical, and post-analytical stages - Selective and cascade antibiotic susceptibility test. Healthcare facility antibiograms: microbiological and sensitivity pattern - Biomarkers |  |  |
| - *Clinical Guidelines I* | - Adaptation of clinical guidelines |  |  |
| - *Clinical Guidelines II* | - Surgical prophylaxis |  |  |
| - *Management of the most frequent infections I* | - Community pneumonia |  |  |
| - *Management of the most frequent infections II* | - Intra-abdominal infection / Urinary tract infection / Management of infections due to multidrug-resistant microorganisms / Skin and soft tissue infection | |  |
| - *Control strategies* | - Restrictive strategies: Pre-authorization. Antibiotic cycling. Auto-stop - Persuasive strategies Prospective audit with feedback - Joint walkthroughs | |  |
| - *Dosing strategies I* | - PK/PD principles. Loading dose. Prolonged infusion - Therapeutic drug monitoring | |  |
| - *Dosing strategies II* | - Debunking myths in the use of antimicrobials - Antimicrobial dosage in special populations: Pregnant women. Obese. Older adults. Critically ill patients | |  |
| - *Pharmacovigilance* | - Strategies for detection of adverse events and prevention of drug-drug interactions - Management of antimicrobial allergy | | |
| - *ASP I metrics* | - Appropriability indicators. Consumption indicators | | |
| - *ASP II Metrics* | - Impact indicators | | |
| - *Information technology and ASP* | - Importance of IT involvement - Areas of collaboration between IT and the ASP - Available tools | | |
| - *Economic analysis* | - Types of health economic analysis - Economic impact analysis. Cost- effectiveness, Cost-benefit, Cost-utility. | | |
| - *Culture and ASP* | - Factors that determine the behavior of prescribers and patients Strategies to positively influence prescribers' and patients' decisions | | |

| **Supplementary - Table 2.** Comparison of scores by domain (means) between for-profit and not-for-profit healthcare facilities | | | | | | | | |
| --- | --- | --- | --- | --- | --- | --- | --- | --- |
| **Domains** |  | **Baseline self-assessment (averages)** | | | |  |  | |
|  | **For-profit**  **(n=29)** | | **Not-for-profit**  **(n=51)** | | **Difference** | **95%CI** | **p***** | |
| Administrative support, PROA Team and Infrastructure | 59·9 | ±18·0 | 52·8 | ±19·1 | 7·1 | -1·62 to 17·72 | 0·1094 | |
| Clinical practice guidelines | 68·8 | ±28·2 | 50·6 | ±27·5 | 18·2 | 5·37 to 31·05 | 0·0060 | |
| Strategies to optimize antimicrobial prescribing· | 67·3 | ±19·3 | 58·2 | ±20·0 | 9·2 | 0·02 to 18·33 | 0·0495 | |
| Monitoring and Reporting | 52·5 | ±20·6 | 49·6 | ±20·9 | 2·9 | -6·69 to 12·55 | 0·5464 | |
| Education, Training and Safety Climate | 42·0 | ±22·0 | 36·6 | ±22·0 | 5·4 | -4·76 to 15·64 | 0·2914 | |
| **Overall score** | **57·7** | **±16·3** | **49·5** | **±16·6** | **8·2** | **0·57 to 15·85** | **0·0345** | |

*Student's *t*-test for independent samples

| **Supplementary - Table 3.** Impact of project participation on knowledge and implementation or changes in antimicrobial stewardship strategies | | | | | |
| --- | --- | --- | --- | --- | --- |
| **Items** | **Top two*** | | **Difference** | **95% CI** | **P** |
|  | **June 2024 (n=80)** | **November 2024 (n=73)** |  |  |  |
| Did participation in the project sessions increase your knowledge of the steps and activities for the development, implementation, and monitoring of an ASP? | 93·8% | 97·3% | 3·5% | 10·1% to -3·1% | 0·2993 |
| Has your participation in previous project sessions served as a basis for implementing or changing specific ASP strategies in your workplace? | 86·3% | 95·9% | 9·6%% | 0·5% to 18·8% | 0·0389 |

* Top two: Definitively yes + Probably yes

**Supplementary - Table 4.** Implementation or changes in antimicrobial stewardship strategies because of participation in the project

| **Items** | **Implemented or modified after project start** | | **Difference** | **95%CI** | **P** |
| --- | --- | --- | --- | --- | --- |
|  | **June 2024 (n=80)** | **November 2024 (n=73)** |  |  |  |
| Implementation or modification of surgical prophylaxis guideline | 18·8% | 41·1% | 22·3% | 8·2% to 36·5% | 0·0043 |
| Implementation or modification of guidelines for the management of common infections | 26·3% | 56·2% | 29·9% | 15·0% to 44·8% | 0·0003 |
| Implementation or modification of guidelines for the management of infections in immunocompromised patients | 10·0% | 17·8% | 7·8% | -3·1% to 18·7% | 0·2433 |
| Implementation or modification of guidelines for the management of infections caused by multidrug-resistant microorganisms | 20·0% | 43·8% | 23·8% | 9·5% to 38·2% | 0·0027 |
| Restrictive control strategies (e.g., surgical prophylaxis hitchhiking; pre-authorization) | 13·8% | 34·2% | 20·5% | 7·3% to 33·7% | 0·0052 |
| Persuasive control strategies (e.g., audits with feedback; joint tours of inpatient wards) | 22·5% | 53·4% | 30·9% | 16·3% to 45·6% | 0·0002 |
| Diagnostic optimization (e.g., rapid diagnostic methods; review of sample collection and transfer; cascade antibiograms; etc…) | 25·0% | 37·0% | 12·0% | -2·6% to 26·6% | 0·1528 |
| pK-pD strategies (e.g., dose adjustment; prolonged infusion; loading dose; etc...) | 8·8% | 34·2% | 25·5% | 13·0% to 38·0% | 0·0002 |
| De-escalation. Transfer to oral route | 17·5% | 34·2% | 16·7% | 3·0% to 30·5% | 0·0286 |
| Healthcare team education and training | 30·0% | 53·4% | 23·4% | 8·2% to 38·6% | 0·0055 |
| Patient and family education on the use of antimicrobials | 7·5% | 23·3% | 15·8% | 4·5% to 27·1% | 0·0123 |
| Measuring the appropriateness of the use of antimicrobial agents | 22·5% | 41·1% | 18·6% | 4·1% to 33·1% | 0·0214 |
| Measuring the consumption of antimicrobials | 16·3% | 37·0% | 20·7% | 7·0% to 34·4% | 0·0063 |
| Measurement of the cost associated with the use of antimicrobials. | 18·8% | 17·8% | -0·9% | -13·2% to 11·3% | 0·9531 |
| Measurement of ASP impact (e.g., incidence density of MMDR infections; incidence density of *C. difficile* infections; mortality, length of hospitalization) | 18·8% | 32·9% | 14·1% | 0·3% to 27·9% | 0·0692 |

**Supplementary - Table 5.** Changes in healthcare personnel practices linked to antimicrobial use.

| **Have there been any changes in the following practices of your healthcare facility's staff personnel (e.g., medical staff, nursing staff) because of your participation in the project sessions?** | | | | | |  |
| --- | --- | --- | --- | --- | --- | --- |
| **Items** | **Top two*** | | **Difference** | **95% CI** | **P** | |
|  | **June 2024 (n=80)** | **November 2024 (n=73)** |  |  |  |  |
| Use antimicrobials only in infections that require it | 55·0% | 80·8% | 25·8% | 11·7% to 40·0% | 0·0012 | |
| Perform cultures before starting antimicrobial treatment when indicated | 50·0% | 61·6% | 11·6% | 27·4% to -4·1% | 0·1988 | |
| Adapt antimicrobial regimens to local epidemiology | 46·3% | 54·8% | 8·5% | 24·4% to -7·3% | 0·3713 | |
| Use narrower-spectrum antibiotics instead of broad- spectrum antibiotics, when appropriate | 31·3% | 49·3% | 18·1% | 33·4% to 2·7% | 0·0345 | |
| Administer antimicrobials at the appropriate time, at the appropriate dose, by the appropriate route | 40·0% | 46·6% | 6·6% | 22·3% to -9·1% | 0·5113 | |
| Consider adverse effects when selecting an antimicrobial regimen for patients | 38·8% | 45·2% | 6·5% | 22·1% to -9·2% | 0·5193 | |
| Confirm history of antimicrobial allergy before changing the regimen | 38·8% | 54·8% | 16·0% | 31·9% to -0·2% | 0·0679 | |
| Discontinue the use of antimicrobials when infection is unlikely | 36·3% | 39·7% | 3·5% | 18·9% to -11·9% | 0·7827 | |
| De-escalate the antimicrobial regimen when the patient's condition permits | 43·8% | 57·5% | 13·8% | 29·6% to -2·1% | 0·1232 | |
| Rotate the antimicrobial to the oral route when the patient's condition and therapeutic options allow it | 43·8% | 45·2% | 1·5% | 17·2% to -14·3% | 0·9856 | |
| Do not prolong the use of antimicrobials beyond what is proven by scientific evidence | 38·8% | 45·2% | 6·5% | 22·1% to -9·2% | 0·5193 | |
| Educate patients/families on the appropriate use of antimicrobials | 46·3% | 42·5% | -3·8% | 11·9% to -19·5% | 0·7584 | |

* Top two: Definitively yes + Probably yes

**Supplementary - Table 6.** Factors identified as enablers factors (73 centers)

| **Factors** | **#** | **%** |
| --- | --- | --- |
| Teamwork | 49 | 16% |
| Institutional support | 37 | 12% |
| Implementation of an improvement process | 27 | 9% |
| Effective communication | 23 | 7% |
| Training of healthcare personnel | 21 | 7% |
| Access to information/technology | 20 | 6% |
| Support from the care staff | 20 | 6% |
| Development/adaptation/access to clinical guidelines | 19 | 6% |
| Staff motivation | 17 | 6% |
| Implementation of monitoring and feedback | 12 | 4% |
| ASP development level | 11 | 4% |
| Microbiology laboratory support | 11 | 4% |
| Available human resources | 8 | 3% |
| Patient safety culture | 7 | 2% |
| Expert support | 5 | 2% |
| Care impact | 5 | 2% |
| Adaptation to change | 5 | 2% |
| Material resources available | 4 | 1% |
| Cost reduction | 3 | 1% |
| Access to antimicrobials | 2 | 1% |
| Existence of government rules and regulations | 2 | 1% |
| Support from scientific societies | 1 | 0% |
| **Total** | **309** | **100%** |

**Supplementary - Table 7.** Factors identified as barriers (73 centers)

| **Factors** | **#** | **%** |
| --- | --- | --- |
| Lack of or limited access to human resources | 38 | 14% |
| Resistance by the healthcare staff to change | 31 | 12% |
| Lack of training of healthcare personnel | 30 | 11% |
| Lack of or limited access to information/technology | 23 | 9% |
| Lack of support from healthcare personnel | 15 | 6% |
| Lack of top management support | 14 | 5% |
| Lack of teamwork | 14 | 5% |
| Staff turnover | 12 | 4% |
| Lack of or limited access to material resources | 12 | 4% |
| Low adherence to clinical guidelines | 11 | 4% |
| Lack of motivation | 11 | 4% |
| Lack of or limited access to antimicrobials | 9 | 3% |
| Complexity of care | 9 | 3% |
| Lack of or limited access to clinical guidelines | 8 | 3% |
| Work burnout | 6 | 2% |
| Lack of awareness of resistance | 5 | 2% |
| Lack of monitoring and feedback | 5 | 2% |
| Deficits in laboratory processes | 5 | 2% |
| Lack of budget | 5 | 2% |
| Lack of patient safety culture | 4 | 1% |
| Lack of government rules or regulations | 2 | 1% |
| **Total** | **269** | **100%** |
